# DNA methylation inhibitors adverse reaction characteristic analysis: an analysis based on the European spontaneous adverse event reporting system

**DOI:** 10.3389/fphar.2024.1527903

**Published:** 2025-01-14

**Authors:** Xia Zhang, Yuyu Liu, Qingwang Hou, Yongxin Guo, Youfu He

**Affiliations:** ^1^ Department of Oncology, Guihang Guiyang Hospital, Guiyang, Guizhou, China; ^2^ School of Integrative Medicine, Nanjing University of Chinese Medicine, Nanjing, Jiangsu, China; ^3^ Department of Cardiology, Henan Provincial People’s Hospital, Hennan University People’s Hospital, Zhengzhou, Henan, China; ^4^ Department of Cardiology, Guizhou Provincial People’s Hospital, Guiyang, Guizhou, China

**Keywords:** DNA methylation inhibitors, adverse drug reactions, system organ class, eudravigilance database, comparative observational study, azacitidine, decitabine

## Abstract

**Introduction:**

DNA methylation inhibitors have been approved for the prevention of Acute Myeloid Leukemia (AML), and their safety profile is not fully characterized. This study was aimed at evaluating the adverse drug reactions (ADRs) of DNA methylation inhibitors by analyzing the individual case safety reports (ICSRs) collected in the EudraVigilance (EV) database.

**Materials and methods:**

The EV database managed by the European Medicines Agency was adopted. The standardized medical terminology set MedDRA was utilized. The ICSRs data of DNA methylation inhibitors for the treatment of acute myeloid leukemia originated from the EV database (2005–2024). A descriptive exploration of the combined data from EV was undertaken to assess the age, gender of patients, severity and outcome of ADR, event year, geographical origin and the qualification of the reporting source. A comprehensive assessment was made for severe ADR cases. By means of the Reporting Odds Ratio (ROR) and 95% Confidence Interval (CI), a non-proportional analysis was made for MedDRA^®^ SOC in DNA methylation inhibitors. Statistical analysis was executed with SPSS version 23.0, and *p* < 0.05 was regarded as statistically significant.

**Result:**

The study reveals that reports related to AZACITIDINE increased from 2005 to 2023, with a slight decline in 2024, while those for DECITABINE have been on the rise since 2007. ICSRs were associated with a majority of males and individuals aged 65–85. Healthcare professionals frequently reported ICSRs related to DNA methylation inhibitors. A significant portion of these ICSRs were serious and completely resolved. The most common ADRs were identified, and certain ADRs had a higher reporting probability with AZACITIDINE (e.g., Febrile neutropenia, Anamia, etc.) and others with DECITABINE (e.g., Myelosuppression, Thrombocytopenia, etc.).

**Conclusion:**

The analysis regarding ADRs of DNA methylation inhibitors was consistent with the literature information disclosed. AZACITIDINE and DECITABINE each have ADRs with a high probability of being reported. Although the study has the advantage of using the database, it is limited by the spontaneous reporting system. Future improvements are needed to accurately evaluate the safety of the drugs.

## Introduction

Acute myeloid leukemia is a malignant disorder of hematopoietic stem cells, which is characterized by the explosive proliferation of myeloid blasts, expansion and differentiation arrest. This leads to ineffective normal hematopoiesis and life-threatening cytopenia and transfusion dependence (DiNardo et al., 2023), along with severe infections, anemia and bleeding (Short et al., 2018). Acute myeloid leukemia can affect individuals of all age groups; it is frequently seen in the elderly, with the median age at diagnosis being 68 years, and over two-thirds of the diagnoses of acute myeloid leukemia occur in patients aged 55 or above (Sasaki et al., 2021).

DNA methylation constitutes a crucial epigenetic modification modality (Marx, 2016). DNA methylation is indispensable to imprinting, X inactivation, and the silencing of pluripotent or tissue-specific genes, thereby governing embryonic development. It mainly acts on gene expression. Under normal conditions, the methylation status of CpG islands in the promoter region of specific genes is appropriate to regulate transcription. When abnormal, excessive methylation of tumor suppressor genes will cause them to be silent, and hypomethylation of proto-oncogenes will lead to their overexpression, increasing the risk of tumors and promoting abnormal proliferation and transformation of cells. Its abnormality will lead to malignant transformation of cells and disrupt the regulation of the cell cycle, cell apoptosis and DNA damage repair mechanisms (Gros et al., 2012). This is the pathogenesis of many diseases, such as neurological disorders, cardiovascular diseases and cancer (Li et al., 2024).

AZACITIDINE and DECITABINE are common DNA methylation inhibitors in recent years and hold significant positions in disease treatment. They are mainly utilized for treating hematological disorders such as myelodysplastic syndrome (MDS) and acute myeloid leukemia (AML) (Sorrentino et al., 2021). After AZACITIDINE is incorporated into DNA within cells, it can form a covalent complex with DNA methyltransferase. Under normal circumstances, DNA methyltransferases are responsible for adding methyl groups to specific regions of DNA. Once they are inactivated, they can no longer catalyze the DNA methylation reaction, thereby preventing the occurrence of DNA methylation. In tumor cells, the promoter regions of many tumor suppressor genes are in a silent state due to excessive methylation and cannot be expressed normally to exert the function of inhibiting the growth of tumor cells. This inhibitory effect enables the re-expression of tumor suppressor genes that were previously silenced due to excessive methylation, restoring their normal cellular regulatory functions and subsequently exerting anti-tumor effects (Sullivan et al., 2005).DECITABINE, on the other hand, irreversibly binds to DNA methyltransferase, causing its consumption and reducing its content within cells. Due to the reduction in the number of DNA methyltransferases, their ability to catalyze DNA methylation also decreases, thereby reducing the overall DNA methylation level of the cells. This allows the expression of some key genes to be restored, alters the biological characteristics of tumor cells, inhibits the growth and survival of tumor cells, induces their differentiation into normal cells or prompts their apoptosis, ultimately achieving the purpose of tumor treatment (Stresemann and Lyko, 2008).

In AML, abnormal DNA methylation is one of the most commonly observed alterations. Recent studies have shown that specific DNA methylation patterns are characteristic of AML. Correspondingly, epigenetic therapies (such as hypomethylating agents) have shown significant activity in AML (Schoofs and Muller-Tidow, 2011).

However, studies centered on ADRs associated with all DNA methylation inhibitors and founded on a spontaneous reporting system (SRS) database are lacking. Several concerns merit discussion. Therefore, this study was aimed at evaluating the ADRs of DNA methylation inhibitors by analyzing the ICSRs collected in the EV database.

## Materials and methods

### Data collection and collation

The EV database, managed by the European Medicines Agency, is employed for collecting and monitoring the data of suspected adverse drug reactions of authorized drugs within the European Economic Area (EEA). It offers valuable information for evaluating the risks and benefits of drugs and guaranteeing public medication safety, encompassing various significant data related to drugs. The EV database is mainly categorized into two principal modules. The Post-authorization Module of EudraVigilance (EVPM) deals with spontaneous reports and non-interventional studies. Its role is to collect and analyze the suspected ADRs that arise during the actual use of drugs after their marketing, facilitating the monitoring of the safety of drugs in widespread applications. The Clinical Trials Module of EudraVigilance (EVCTM) concentrates on adverse drug reaction reports related to interventional studies. This module is beneficial for evaluating the possible adverse reactions of drugs in a strictly controlled clinical trial environment, furnishing a basis for drug approval and regulatory decisions.

The International Council for Harmonisation of Technical Requirements for Pharmaceuticals for Human Use (ICH) develops and maintains MedDRA, a standardized medical terminology set that is widely applied in global drug regulatory affairs, with the intention of facilitating the consistency, accuracy, and clarity of data in drug research and development and regulation. It covers various medical terms such as symptoms, signs, disease diagnoses, etc. It has a multi-level structure including SOC, High-Level Term (HLT), Preferred Term (PT), etc., which enables more precise and detailed encoding and classification of medical information.

The data of ICSRs for DNA methylation inhibitors (identified as suspected drugs) used for the treatment of acute myeloid leukemia originated from the EV database (accessed on 25 October 2024), covering the relevant data from 2005 to 2024, including all ICSR cases of azacitidine and decitabine recorded from the drug approval time to 2024. All pre-market ICSRs with supporting literature data were excluded before conducting further analysis. Additionally, to prevent treatment bias, all ICSRs with other indications and more than one reported suspected drug were also eliminated. The selection for the analysis was determined based on the use of the SOC of the Medical Dictionary for Regulatory Activities (MedDRA^®^) of “Cardiac Disorders” or “Blood and Lymphatic System Disorders”.

This study utilized a public database. To ensure that the research met ethical standards, we took the following measures: Firstly, the obtained data were strictly anonymized to protect the privacy of data providers. Secondly, we confirmed the usage license of the database employed to guarantee that our research activities were conducted within a legal and compliant framework.

### Descriptive analysis

A descriptive analysis was conducted on the aggregated data from EV to evaluate the following criteria: the age and gender of patients, ADR information (severity and outcome), the year of the event, geographical origin, and the qualifications of the primary reporting source. According to the International Council for Harmonisation E2D guidelines, ADRs are classified as severe under specific conditions: if they are considered as death or life-threatening situations, if they are considered as cases of hospitalization or prolongation of existing hospital stays, if they are considered as cases of persistent or severe disability/incapacity, if they are considered as cases of congenital anomalies or defects, or if they are included in the list of Important Medical Events (IME) updated by the European Medicines Agency based on MedDRA^®^ every 6 months. The outcomes of ADRs are classified as “recovered/resolved”, “recovering/resolving”, “recovered/resolved with sequelae”, “not recovered/not resolved”, and “fatal”. If there are two or more ADRs with different outcomes in a single ICSR, then the outcome with the lowest resolution should be picked out and used for classification.

### Comprehensive assessment of severe ADRs cases

A comprehensive assessment was conducted on severe ADR cases, with a focus on severity criteria (life-threatening, disabling, and fatal).

### Disproportionate analysis

Furthermore, a disproportionate analysis was carried out using the ROR and the corresponding 95% CI to evaluate the reporting frequency of ADRs for MedDRA^®^ SOC in DNA methylation inhibitors. The reference group included all DNA methylation inhibitors except the one of interest. If the total number of cases reached or exceeded three, the ROR and 95% CI were evaluated.

### Statistical software and significance determination

Statistical analysis was carried out using version 23.0 of the Statistical Package for the SPSS for Windows (provided by IBM SPSS Statistics). All analyses conducted through SPSS were regarded as statistically significant if the *p*-value was less than 0.05.

## Result attributes of ICSRs

**FIGURE 1:**
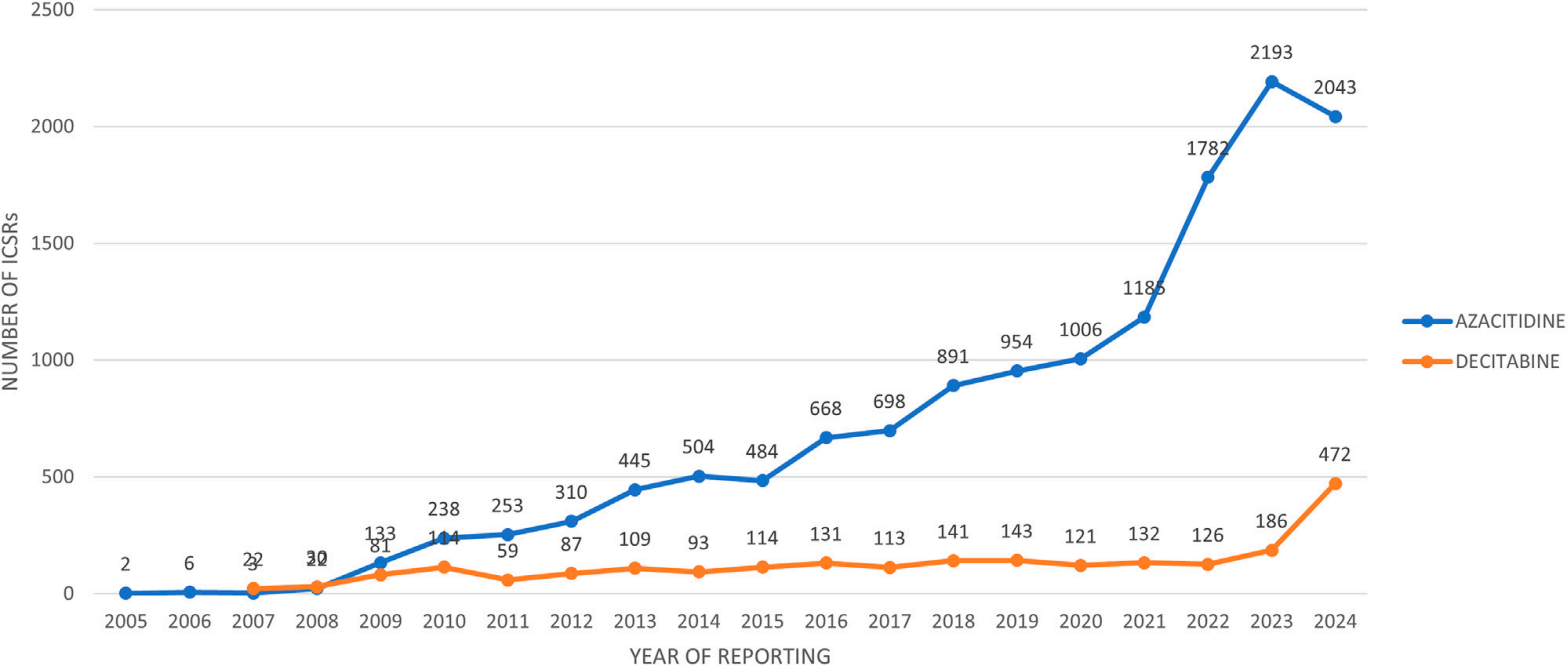
Trend over the years of AZACITIDINE and DECITABINE, ICSRs individual case safety reports.

The trend over the years shows that the reports related to AZACITIDINE continued to increase from 2005 to 2023, until there was a small decline in 2024. While the reports related to DECITABINE have been continuously increasing from 2007 to 2024 (Figure 1).

Substantially, ICSRs were associated with females (37.5%), males (55.4%), and individuals aged 65–85 years (52.6%). Healthcare professionals reported ICSRs related to DNA methylation inhibitors at a higher frequency (90.3%). A greater proportion of DNA methylation inhibitors-related ICSRs were serious and completely resolved (n = 7559; 47.0%) (Table 1).

**TABLE 1:** Characteristics of ICSRs for DNA methylation inhibitors reported in EV.

Characteristic, n (%)	AZACITIDINE (13820)	DECITABINE (2274)	Total (16094)
Age group			
Not Specified	2498 (18.1)	565 (24.8)	3063 (19.0)
0–17 Years	256 (1.9)	83 (3.6)	339 (2.1)
18–64 Years	3265 (23.6)	813 (35.8)	4078 (25.3)
65–85 Years	7271 (52.6)	774 (34.0)	8045 (50.0)
More than 85 Years	530 (3.8)	39 (1.7)	569 (3.5)
Patient sex			
Female	5185 (37.5)	844 (37.1)	6029 (37.5)
Male	7652 (55.4)	1262 (55.5)	8914 (55.4)
Not Specified	983 (7.1)	168 (7.4)	1151 (7.2)
Primary source qualification			
Healthcare Professional	12483 (90.3)	2115 (93.0)	14598 (90.7)
Non Healthcare Professional	1335 (9.7)	159 (7.0)	1494 (9.3)
Not Specified			
Primary source country for regulatory purposes			
European Economic Area	4383 (31.7)	292 (12.8)	4675 (29.0)
Non European Economic Area	9437 (68.3)	1982 (87.2)	11419 (71.0)
Serious	18680	3662	22342
Type of seriousness			
Other Medically Important Condition	9948 (72.0)	2403 (105.7)	12351 (76.7)
Caused/Prolonged Hospitalisation	6345 (45.9)	820 (36.1)	7165 (44.5)
Results in Death	1862 (13.5)	367 (16.1)	2229 (13.8)
Life Threatening	371 (2.7)	48 (2.1)	419 (2.6)
Disabling	154 (1.1)	24 (1.1)	178 (1.1)
Outcome			
Recovered/Resolved	6642 (48.1)	917 (40.3)	7559 (47.0)
Recovering/Resolving	3582 (25.9)	745 (32.8)	4327 (26.9)
Recovered/Resolved With Sequelae	236 (1.7)	8 (0.4)	244 (1.5)
Not Recovered/Not Resolved	3723 (26.9)	476 (20.9)	4199 (26.1)
Fatal	3838 (27.8)	610 (26.8)	4448 (27.6)

The most commonly reported ADRs were Febrile neutropenia (n = 1738; 18.8%), Anemia (n = 826; 8.9%), Cytopenia (n = 372; 4.0%), Bone marrow failure (n = 91; 1.0%), Pericarditis (n = 74; 0.8%), Cardiac disorder (n = 42; 0.5%), Myelosuppression (n = 1950; 21.1%), Thrombocytopenia (n = 997; 10.8%), Leukopenia (n = 466; 5%), and Hematotoxicity (n = 158; 1.7%). With AZACITIDINE, a higher likelihood of reporting was demonstrated for Febrile neutropenia (ROR = 1.27; 95%CI = 1.10–1.47), Anamia (1.49; 1.20–1.86), Cytopenia (1.49; 1.07–2.07), Bone marrow failure (2.54; 1.11–5.81), Pericarditis (4.42; 1.39–14.03), and Cardiac disorder (7.52; 1.04–54.69), while with DECITABINE, it was for Myelosuppression (1.46; 1.30–1.63), Thrombocytopenia (1.21; 1.03–1.43), Leukopenia (4.44; 3.69–5.34), and Hematotoxicity (4.15; 3.02–5.70; Table 2).

**TABLE 2:** ROR of ICSRs with ADRs belonging to the SOC “Blood and lymphatic system disorders” or “Cardiac disorders” via PT for the comparison of DNA methylation inhibitors.

SOC	PT	AZACITIDINE		DECITABINE		Total
		N	ROR (95% CI)	N	ROR (95% CI)	N
Blood and lymphatic system disorders						
	Myelosuppression	1554	0.69 (0.61–0.77)	396	**1.46 (1.30–1.63)**	1950
	Febrile neutropenia	1522	**1.27 (1.10–1.47)**	216	0.78 (0.68–0.91)	1738
	Neutropenia	1094	0.86 (0.74–1.00)	226	1.16 (1.00–1.34)	1320
	Thrombocytopenia	820	0.82 (0.70–0.97)	177	**1.21 (1.03–1.43)**	997
	Anemia	737	**1.49 (1.20–1.86)**	89	0.67 (0.54–0.84)	826
	Pancytopenia	446	1.01 (0.79–1.29)	79	0.99 (0.78–1.26)	525
	Cytopenia	332	**1.4 (1.07–2.07)**	40	0.67 (0.48–0.93)	372
	Leukopenia	263	0.23 (0.19–0.27)	203	**4.4 (3.69–5.34)**	466
	Agranulocytosis	95	0.65 (0.42–1.01)	26	1.53 (0.99–2.37)	121
	Hemototoxicity	91	0.24 (0.18–0.33)	67	**4.1 (3.02–5.70)**	158
	Bone marrow failure	85	**2.5 (1.11–5.81)**	6	0.39 (0.17–0.90)	91
	Leukocytosis	39	0.78 (0.38–1.60)	9	1.29 (0.62–2.66)	48
Cardiac disorders	Cardiac failure	123	1.05 (0.66–1.67)	21	0.95 (0.60–1.52)	144
	Atrial fibrillation	100	1.38 (0.77–2.46)	13	0.73 (0.41–1.29)	113
	Pericarditis	74	**4.4 (1.39–14.03)**	—	—	74
	Cardiac disorder	42	**7.5 (1.04–54.69)**	—	—	42
	Cardiac arrest	38	0.97 (0.43–2.18)	7	1.03 (0.46–2.31)	45
	Pericardial effusion	37	1.66 (0.59–4.65)	4	0.60 (0.22–1.69)	41
	Tachycardia	32	1.43 (0.51–4.05)	4	0.70 (0.25–1.98)	36
	Myocardial infarction	32	0.72 (0.33–1.55)	8	1.40 (0.64–3.03)	40
	Cardiac failure congestive	26	0.78 (0.32–1.88)	6	1.29 (0.53–3.14)	32
	Cardiac failure acute	19	3.40 (0.46–25.42)	—	—	19
	Arrhythmia	18	0.64 (0.24–1.74)	5	1.55 (0.58–4.18)	23
	Acute myocardial infarction	18	0.64 (0.24–1.74)	5	1.55 (0.58–4.18)	23

ICSR, individual case safety report; PT, preferred term; ROR, reporting odds ratio; SOC, system organ class Significant RORs are in bold type.

We conducted an in-depth study on the top 20 ADRs reported for each DNA methylation inhibitor in the SOCs, and a total of 152 identical signals were found in the PTs of the two inhibitors. All common signals were sorted and recorded in Table 3. Among them, the SOC with the most adverse signals was General disorders and administration site conditions, and the top five were Death, Condition aggravated, Drug Intolerance, Disease progression, and Mucosal inflammation. Next was Infections and infestations, and the top five were Infection, Sinusitis, Neutropenic sepsis, Urinary tract infection, and Staphylococcal infection.

**TABLE 3:** Same ADRs among two DNA methylation inhibitors.

System organ classes	ADRs	Signal N
Blood and lymphatic system disorders	Myelosuppression, Cytopenia, Neutropenia, Febrile neutropenia, Leukopenia, Pancytopenia, Bone marrow failure, Anemia, Splenomegaly, Disseminated intravascular coagulation, Thrombocytosis, Leukocytosis, Haematotoxicity, Thrombocytopenia, Agranulocytosis	10
Cardiac disorders	Tachycardia, Cardiac failure, Atrial fibrillation, Arrhythmia, Cardiac arrest, Myocardial infarction, Pericarditis, Pericardial effusion, Cardiac failure congestive, Acute myocardial infarction	10
Gastrointestinal disorders	Abdominal pain, Abdominal distension, Colitis, Gastrointestinal disorder, Ascites, Gingival bleeding, Abdominal pain upper, Nausea, Vomiting, Stomatitis, Diarrhoea, Gastrointestinal haemorrhage, Constipation	13
General disorders and administration site conditions	Death, Condition aggravated, Drug intolerance, Disease progression, Mucosal inflammation, General physical health deterioration, Pyrexia, Fatigue, Chest pain, Multiple organ dysfunction syndrome, Therapeutic product effect incomplete, Disease recurrence, Pain, Chills, Therapeutic response decreased, Drug ineffective, Treatment failure, Drug interaction, Oedema peripheral, Malaise, Asthenia	20
Hepatobiliary disorders	Hepatic function abnormal, Liver disorder, Hyperbilirubinaemia, Hepatic failure	4
Immune system disorders	Hypersensitivity, Acute graft versus host disease, Immunodeficiency, Chronic graft versus host disease, Graft versus host disease	5
Infections and infestations	Infection, Sinusitis, Neutropenic sepsis, Urinary tract infection, Staphylococcal infection, *Clostridium difficile* colitis, Upper respiratory tract infection, Bacteraemia, Bronchopulmonary aspergillosis, Pneumonia, Septic shock, Respiratory tract infection, Pneumonia bacterial, Sepsis, Pneumonia fungal, Cellulitis, Diverticulitis, Aspergillus infection, Fungal infection	19
Injury, poisoning and procedural complications	Fall, Product use in unapproved indication, Intentional product use issue, Product use issue, Toxicity to various agents, Contusion, Off label use	7
Metabolism and nutrition disorders	Hypokalaemia, Tumourlysis syndrome, Decreased appetite, Dehydration, Hyponatraemia	5
Musculoskeletal and connective tissue disorders	Back pain, Arthritis, Pain in extremity, Myalgia, Arthralgia	5
Neoplasms benign, malignant and unspecified (incl cysts and polyps)	Malignant neoplasm progression, Chronic myelomonocytic leukaemia, Leukaemia, Leukaemia recurrent, Acute myeloid leukaemia refractory, Acute myeloid leukaemia recurrent, Differentiation syndrome, Neoplasm progression, Acute myeloid leukaemia, Acute leukaemia, Myelodysplastic syndrome	11
Nervous system disorders	Loss of consciousness, Somnolence, Neuropathy peripheral, Haemorrhage intracranial, Dizziness, Seizure, Headache, Cerebral haemorrhage	8
Psychiatric disorders	Confusional state	1
Renal and urinary disorders	Renal failure, Acute kidney injury, Renal impairment, Haematuria	4
Respiratory, thoracic and mediastinal disorders	Dyspnoea, Cough, Acute respiratory failure, Epistaxis, Pleural effusion, Pulmonary oedema, Respiratory failure, Organising pneumonia, Acute respiratory distress syndrome, Respiratory distress, Pulmonary embolism, Pneumonitis, Pulmonary haemorrhage, Hypoxia, Lung disorder, Interstitial lung disease, Lung infiltration	16
Skin and subcutaneous tissue disorders	Pruritus, Rash pruritic, Rash, Acute febrile neutrophilic dermatosis, Erythema, Petechiae, Rash erythematous, Skin exfoliation, Alopecia	9
Vascular disorders	Deep vein thrombosis, Haemorrhage, Hypotension, Hypertension, Haematoma	5

When comparing the top 20 ADRs of the two drugs in the SOCs, we found that there were differences in the PTs of many ADRs between the two inhibitors, such as Blood and lymphatic system disorders, Cardiac disorders, Gastrointestinal disorders, etc. (Table 4). The respective numbers of unique symptoms for AZACITIDINE and DECITABINE were 70 and 84.

**TABLE 4:** Different ADRs among two DNA methylation inhibitors.

System organ classes	AZACITIDINE	DECITABINE
	Platelet disorder, Blood disorder, Febrile bone marrow aplasia	Bicytopenia, Granulocytopenia
Cardiac disorders	Cardiac disorder, Angina pectoris, Cardiac failure acute	Palpitations, Cardiomyopathy, Left ventricular dysfunction
	Haematemesis, Dyspepsia, Pancreatitis acute, Rectal haemorrhage, Dysphagia, Neutropenic colitis, Ileus, Melaena	Haematochezia, Mouth ulceration, Oral pain, Intestinal obstruction, Proctalgia
General disorders and administration site conditions	Injection site haematoma, Injection site pruritus, Injection site erythema, Injection site reaction, Unevaluable event, Application site pain, Injection site pain, Adverse event, Application site erythema	Drug resistance, Swelling face, Peripheral swelling, Drug ineffective for unapproved indication, Extravasation, Hyperpyrexia, Chest discomfort, Therapeutic response increased, Oedema
Hepatobiliary disorders	Venoocclusive liver disease, Jaundice	Liver injury
	Haemophagocytic lymphohistiocytosis, Immune system disorder	
Infections and infestations	Pneumocystis jirovecii pneumonia, *Pseudomonas* infection, Skin infection, Staphylococcal bacteraemia, Necrotising fasciitis, Enterococcal infection, Lower respiratory tract infection, Influenza, Bronchitis, Bacterial infection, Staphylococcal sepsis	Oral candidiasis, Mucormycosis, Soft tissue infection, Herpes simplex, *Escherichia* bacteraemia, Herpes zoster, Gastrointestinal infection, Cytomegalovirus infection, Cytomegalovirus viraemia, Appendicitis, *Clostridium difficile* infection
Injury, poisoning and procedural complications	Subdural haematoma, Infusion related reaction	Incorrect dose administered, Product prescribing error, Inappropriate schedule of product administration, Product administered to patient of inappropriate age
	Neutrophil count abnormal, Haemoglobin abnormal, Blood lactate dehydrogenase increased, Serum ferritin increased, White blood cell count abnormal, Platelet count abnormal	Transaminases increased, Weight increased, Blast cells present, Blood alkaline phosphatase increased, Liver function test abnormal, Breath sounds abnormal, Blood calcium decreased, Body temperature increased, Blood glucose increased, Blood albumin decreased
	Hyperkalaemia	Cachexia, Hyperuricaemia, Hyperglycaemia, Electrolyte imbalance, Hypoalbuminaemia, Hypophosphataemia
	Minimal residual disease, Myelodysplastic syndrome transformation, Myelofibrosis, Transformation to acute myeloid leukaemia	Chloroma, Blast crisis in myelogenous leukaemia
Nervous system disorders	Syncope, Cerebrovascular accident	Hypoaesthesia, Haemorrhagic stroke, Memory impairment, Dizziness postural, Lethargy, Tremor, Balance disorder, Paraesthesia, Posterior reversible encephalopathy syndrome, Peroneal nerve palsy, Polyneuropathy
	Insomnia, Depression, Delirium	Mental status changes, Depressed mood
	Urinary retention, Renal disorder	Renal tubular necrosis, Cystitis haemorrhagic
	Haemoptysis, Pulmonary alveolar haemorrhage, Dyspnoea exertional	Productive cough, Choking, Oropharyngeal pain, Pulmonary fibrosis
	Pyoderma gangrenosum, Skin lesion, Skin necrosis, Skin reaction, Neutrophilic dermatosis, Urticaria	Hyperhidrosis, Ecchymosis, Panniculitis
Social circumstances	Blood product transfusion dependent	
Vascular disorders	Thrombosis, Shock	Vasculitis, Phlebitis

## Discussion

As far as our knowledge extends, this is the inaugural study that investigates the ADRs associated with DNA methylation inhibitors by means of the analysis of the EV database. DNA methylation inhibitors are of great significance in the field of clinical treatment (Zhang et al., 2024), especially in tumor therapy. They can inhibit DNA methyltransferases, demethylate and restore the expression of tumor suppressor genes, thereby restricting the proliferation of tumor cells and inducing differentiation and apoptosis. They can also upregulate differentiation genes, enhance chemotherapy sensitivity, and activate the body’s immunity to fight tumors. Ever since the emergence of DNA methylation inhibitors, the therapeutic panorama of acute myeloid leukemia has undergone a radical transformation (Sestakova et al., 2022). The rapid onset of action, outstanding patient response and favorable safety profile render DNA methylation inhibitors treatment the premier therapeutic option for acute myeloid leukemia (Li et al., 2016; Bullinger et al., 2010; Issa et al., 2015). They can reactivate tumor suppressor genes that have been silenced by abnormal methylation. In AML, some key tumor suppressor genes will lose their function due to excessive DNA methylation (Yang et al., 2019; Perez et al., 2013). DNA methylation inhibitors can reverse this process and restore the normal expression of these genes, thereby exerting the effects of inhibiting tumor cell growth and promoting cell apoptosis (Uddin and Fandy, 2021). Moreover, DNA methylation inhibitors contribute to altering the epigenetic state of leukemia cells, enabling the cells to regain sensitivity to other therapeutic approaches (Cheng et al., 2019).This implies that they can be combined with traditional chemotherapeutic drugs or targeted therapeutic drugs to enhance the overall therapeutic effect and increase the remission rate and survival rate (Das, 2018). In this study, ICSRs related to DNA methylation inhibitors presented certain characteristics. For instance, the trend of reports indicated that the reports of AZACITIDINE continuously increased from 2005 to 2023 and slightly declined in 2024; the reports of DECITABINE have been on the rise since 2007. Regarding the gender and age distribution, ICSRs mainly involved males (55.4%) and individuals aged 65–85 (52.6%). Healthcare professionals had a higher reporting frequency (90.3%). These data reflect the occurrence of adverse reactions in the practical application of DNA methylation inhibitors and are of great significance for evaluating their safety.

The analysis found that men and the 65–85 age group had a relatively high proportion in ICSRs. The possible reasons are that the incidence of AML is higher in the elderly population (Abdallah et al., 2020), and men in this age group may be more likely to fall ill or receive relevant treatments, thereby resulting in a relatively large number of reports (Short et al., 2018). Additionally, differences in drug metabolism among different genders and age groups may also affect the occurrence and reporting of adverse reactions (LeBlanc et al., 2024). The high proportion of serious ICSRs indicates that the adverse reactions of DNA methylation inhibitors cannot be ignored. This may be related to the severity of the disease and the poor basic health status of AML patients themselves. The ADRs with a higher reporting probability for AZACITIDINE include febrile neutropenia, anemia, cytopenia, bone marrow failure, pericarditis, and cardiac disorders, etc. Febrile neutropenia may be related to the inhibition of the drug on the hematopoietic function of the bone marrow, resulting in a decrease in neutrophil production and thereby increasing the risk of infection (Patel and West, 2017). The occurrence of these ADRs may adversely affect the treatment process of patients, such as increasing the risk of infection, reducing the quality of life, and affecting treatment compliance, etc. The ADRs with a higher reporting probability for DECITABINE are myelosuppression, thrombocytopenia, leukopenia, and hematotoxicity, etc. Myelosuppression is a common adverse reaction of such drugs, which affects the production of various blood cells in the bone marrow, resulting in a decrease in the number of peripheral blood cells (Nian et al., 2024). Thrombocytopenia may increase the risk of bleeding in patients, and leukopenia makes patients more prone to infection.

Studies have shown that both AZACITIDINE and DECITABINE have relatively high reporting probabilities of ADRs, which are of great reference value for clinicians’ initial drug selection. For example, when patients with poor basic conditions and high infection risk use AZACITIDINE, special attention should be paid to ADRs such as febrile neutropenia and anemia, and strengthened monitoring and preventive supportive treatment should be adopted; when patients with good bone marrow reserve function but poor tolerance to hematological toxicity use DECITABINE, attention should be focused on ADRs such as bone marrow suppression and thrombocytopenia, and blood transfusion support should be planned in advance if necessary. The key to reducing the occurrence of ADRs lies in personalized adjustment of drug doses based on individual characteristics of patients (age, weight, physical condition, gene mutation status, etc.). For elderly patients or those with liver and kidney dysfunction, the initial dose should be appropriately reduced due to the possible decrease in their ability to metabolize and excrete drugs, and then gradually adjusted according to the patient’s tolerance and treatment response. During treatment, closely monitor the treatment response and ADRs. When ADRs occur, reduce or suspend the drug in a timely manner according to the severity, and resume carefully after the ADRs are relieved, maintaining at a lower dose or adjusting the plan.

This study offers an overview regarding the safety of DNA methylation inhibitors, and the utilization of the EV database presents considerable advantages. This database is capable of collecting a large amount of real-world ICSRs data, which is helpful for discovering rare or delayed ADRs and providing more comprehensive information for drug safety assessment. Especially for newly approved or less frequently used drugs, this database-based analysis can provide early safety signals and offer important references for the subsequent development, regulatory decisions, and clinical applications of drugs. In future studies, ADR management is indispensable, and its importance is reflected in many aspects: it can not only alleviate adverse reactions such as anemia and gastrointestinal discomfort to ensure the continuity of treatment, but also flexibly adjust the plan according to the patient’s condition; it not only focuses on improving the quality of life of patients to help them receive treatment in a good physical and mental state, but also reduces the risk of complications such as infection and bleeding, comprehensively helping to improve the treatment effect.

Through the analysis of a large amount of data, we can describe more accurately the characteristics of adverse reactions of DNA methylation inhibitors in different populations, providing a basis for individualized treatment. However, this study also has certain limitations. The spontaneous reporting system itself has some inherent problems. For example, data missing may lead to incomplete partial information, affecting the comprehensive assessment of adverse reactions; report duplication may cause data redundancy, interfering with the judgment of the true incidence rate; the lack of the denominator (i.e., the total number of patients with acute myeloid leukemia who have received treatment) makes it impossible for us to accurately calculate the incidence rate of adverse reactions. We can only assess the relative frequency through methods such as the ROR, which has certain limitations. Additionally, there may be underreporting phenomena. Some mild or atypical adverse reactions may not be reported, thereby underestimating the actual adverse reaction risk of the drug. In terms of sample size, although a certain number of ICSRs have been collected, the analysis of some rare adverse reactions may still be insufficient. The study’s time range may also have an impact on the results. For instance, over time, the usage of drugs and patient characteristics may change, and these changes may not be fully covered within the time span of this study. The analysis method (such as ROR analysis) also has its limitations. It can only indicate the degree of association between the drug and adverse reactions and cannot determine the causal relationship. Further studies are needed for verification. Future studies can consider integrating multiple data sources, improving data collection methods, expanding the sample size, extending the study time, and adopting more advanced analysis methods to overcome these limitations and assess the safety of DNA methylation inhibitors more accurately.

Although the current research indicates that DNA methylation inhibitors (such as AZACITIDINE and DECITABINE) have certain therapeutic effects in the treatment of acute myeloid leukemia (AML), single-drug therapy has limitations. Subsequent studies can focus on exploring the optimal regimens for combination use with other new targeted drugs (such as FLT3 inhibitors, IDH inhibitors, etc.), including drug combinations, dose ratios, administration sequences, and treatment cycles. The safety and efficacy of combination therapy can be evaluated through large-scale clinical trials to improve the remission rate and survival rate of patients. Additionally, as the treatment progresses, some patients may develop resistance to DNA methylation inhibitors. In the future, in-depth research can be conducted on the molecular mechanisms related to drug resistance, such as mutations in DNA methyltransferase genes, changes in epigenetic modifications, and the influence of the tumor microenvironment, to find new targets and strategies to overcome drug resistance, and to develop targeted resistance-reversal agents or new therapeutic drugs to improve the treatment effect of patients with drug resistance. When using DNA methylation inhibitors in individualized therapy, first, the gene mutation status is crucial for the selection and use of DNA methylation inhibitors. Different gene mutations may affect the sensitivity and reactivity of tumor cells to the drugs. For example, if a patient has specific gene mutations closely related to DNA methylation, it may be necessary to adjust the dose of the inhibitor or select a specific type of inhibitor. Age is also a key factor. For young patients, their physical functions are usually better and they may be able to tolerate higher doses or more intensive treatment regimens. However, elderly patients may need to reduce the drug dose due to reasons such as organ function decline and decreased metabolic capacity to avoid serious side effects. Comorbidities also affect treatment decisions. If the patient has comorbidities such as cardiovascular diseases, liver and kidney function disorders at the same time, the metabolism and excretion of the drug may be affected. Therefore, it is necessary to carefully select the dose and treatment cycle, and closely monitor the adverse reactions of the drug.

## Conclusion

The analysis of ADRs related to DNA methylation inhibitors was in accordance with the information reported in the literature. The reports in the study were mostly submitted by healthcare professionals. The proportion of serious ICSRs cannot be ignored, and AZACITIDINE and DECITABINE each have ADRs with a high probability of being reported. For AZACITIDINE, the ADRs with a higher reporting probability are febrile neutropenia, anemia, cytopenia, bone marrow failure, pericarditis, and cardiac disorders. For DECITABINE, they are myelosuppression, thrombocytopenia, leukopenia, and hematotoxicity. Although the study benefits from the utilization of the database, it is constrained by the spontaneous reporting system. Future enhancements are requisite to precisely assess the safety of the drugs. In practical applications, doctors need to comprehensively consider all the various factors we mentioned earlier. The gene mutation status of the patient can be clarified through genetic testing, and the overall health status of the patient can be evaluated in combination with age and comorbidities. Before the start of treatment, an individualized treatment plan should be formulated, including the initial dose, treatment interval and expected treatment cycle. During the treatment process, closely monitor the patient’s response, such as symptom improvement and changes in blood indicators, and adjust the treatment plan in a timely manner according to the monitoring results to achieve the best treatment effect and the least adverse reactions.

## Data Availability

The data used in this study were obtained from EudraVigilance (https://www.ema.europa.eu), the European database for adverse drug reaction reports, managed by the European Medicines Agency (EMA). The authors acknowledge the EMA for providing access to the data and emphasize that the interpretations and conclusions presented in this article do not necessarily reflect the views of the EMA. The original contributions presented in the study are included in the article/supplementary material, further inquiries can be directed to the corresponding author.

## References

[B1] Abdallah M. Xie Z. Ready A. Manogna D. Mendler J. H. Loh K. P. (2020). Management of acute myeloid leukemia (AML) in older patients. Curr. Oncol. Rep. 22 (10), 103. 10.1007/s11912-020-00964-1 32725515 PMC8026174

[B2] Bullinger L. Ehrich M. Döhner K. Schlenk R. F. Döhner H. Nelson M. R. (2010). Quantitative DNA methylation predicts survival in adult acute myeloid leukemia. Blood 115 (3), 636–642. 10.1182/blood-2009-03-211003 19903898

[B3] Cheng Y. Wang M. Ma X. Mo F. Yang S. (2019). Targeting epigenetic regulators for cancer therapy: mechanisms and advances in clinical trials. Signal Transduct. Target Ther. 4, 62. 10.1038/s41392-019-0095-0 31871779 PMC6915746

[B4] Das M. (2018). Venetoclax with decitabine or azacitidine for AML. Lancet Oncol. 19 (12), e672. 10.1016/S1470-2045(18)30824-6 30392809

[B5] DiNardo C. D. Erba H. P. Freeman S. D. Wei A. H. (2023). Acute myeloid leukaemia. Lancet 401 (10393), 2073–2086. 10.1016/S0140-6736(23)00108-3 37068505

[B6] Gros C. Fahy J. Halby L. Dufau I. Erdmann A. Gregoire J. M. (2012). DNA methylation inhibitors in cancer: recent and future approaches. Biochimie 94 (11), 2280–2296. 10.1016/j.biochi.2012.07.025 22967704

[B7] Issa J. J. Roboz G. Rizzieri D. Jabbour E. Stock W. O'Connell C. (2015). Safety and tolerability of guadecitabine (SGI-110) in patients with myelodysplastic syndrome and acute myeloid leukaemia: a multicentre, randomised, dose-escalation phase 1 study. Lancet Oncol. 16 (9), 1099–1110. 10.1016/S1470-2045(15)00038-8 26296954 PMC5557041

[B8] LeBlanc F. R. Breese E. H. Burns K. C. Chang E. K. Jones L. M. Lee L. (2024). Clinical outcomes of hypomethylating agents and venetoclax in newly diagnosed unfit and relapsed/refractory paediatric, adolescent and young adult acute myeloid leukaemia patients. Br. J. Haematol. 205 (3), 1055–1066. 10.1111/bjh.19679 39082439

[B9] Li L. Chen R. Zhang H. Li J. Huang H. Weng J. (2024). The epigenetic modification of DNA methylation in neurological diseases. Front. Immunol. 15, 1401962. 10.3389/fimmu.2024.1401962 39376563 PMC11456496

[B10] Li X. Zhu L. Ye X. (2016). Aberrant DNA methylation and its targeted therapy in acute myeloid leukemia. Zhejiang Da Xue Xue Bao Yi Xue Ban. 45 (4), 387–394. 10.3785/j.issn.1008-9292.2016.07.09 27868412 PMC10397039

[B11] Marx V. (2016). Genetics: profiling DNA methylation and beyond. Nat. Methods 13 (2), 119–122. 10.1038/nmeth.3736 26820544

[B12] Nian Q. Liu R. Zeng J. (2024). Unraveling the pathogenesis of myelosuppression and therapeutic potential of natural products. Phytomedicine 132, 155810. 10.1016/j.phymed.2024.155810 38905848

[B13] Patel K. West H. J. (2017). Febrile neutropenia. JAMA Oncol. 3 (12), 1751. 10.1001/jamaoncol.2017.1114 28750112

[B14] Perez C. Pascual M. Martín-Subero J. I. Bellosillo B. Segura V. Delabesse E. (2013). Aberrant DNA methylation profile of chronic and transformed classic Philadelphia-negative myeloproliferative neoplasms. Haematologica 98 (9), 1414–1420. 10.3324/haematol.2013.084160 23716560 PMC3762098

[B15] Sasaki K. Ravandi F. Kadia T. M. DiNardo C. D. Short N. J. Borthakur G. (2021). *De novo* acute myeloid leukemia: a population-based study of outcome in the United States based on the Surveillance, Epidemiology, and End Results (SEER) database, 1980 to 2017. Cancer 127 (12), 2049–2061. 10.1002/cncr.33458 33818756 PMC11826308

[B16] Schoofs T. Muller-Tidow C. (2011). DNA methylation as a pathogenic event and as a therapeutic target in AML. Cancer Treat. Rev. 37 (Suppl. 1), S13–S18. 10.1016/j.ctrv.2011.04.013 21612874

[B17] Sestakova S. Cerovská E. Šálek C. Kundrát D. Ježíšková I. Folta A. (2022). A validation study of potential prognostic DNA methylation biomarkers in patients with acute myeloid leukemia using a custom DNA methylation sequencing panel. Clin. Epigenetics 14 (1), 22. 10.1186/s13148-022-01242-6 35148810 PMC8832751

[B18] Short N. J. Rytting M. E. Cortes J. E. (2018). Acute myeloid leukaemia. Lancet 392 (10147), 593–606. 10.1016/S0140-6736(18)31041-9 30078459 PMC10230947

[B19] Sorrentino V. G. Thota S. Gonzalez E. A. Rameshwar P. Chang V. T. Etchegaray J. P. (2021). Hypomethylating chemotherapeutic agents as therapy for myelodysplastic syndromes and prevention of acute myeloid leukemia. Pharm. (Basel) 14 (7), 641. 10.3390/ph14070641 PMC8308509 34358067

[B20] Stresemann C. Lyko F. (2008). Modes of action of the DNA methyltransferase inhibitors azacytidine and decitabine. Int. J. Cancer 123 (1), 8–13. 10.1002/ijc.23607 18425818

[B21] Sullivan M. Hahn K. Kolesar J. M. (2005). Azacitidine: a novel agent for myelodysplastic syndromes. Am. J. Health Syst. Pharm. 62 (15), 1567–1573. 10.2146/ajhp040385 16030365

[B22] Uddin M. G. Fandy T. E. (2021). DNA methylation inhibitors: retrospective and perspective view. Adv. Cancer Res. 152, 205–223. 10.1016/bs.acr.2021.03.007 34353438 PMC10275377

[B23] Yang X. Wong M. P. M. Ng R. K. (2019). Aberrant DNA methylation in acute myeloid leukemia and its clinical implications. Int. J. Mol. Sci. 20 (18), 4576. 10.3390/ijms20184576 31527484 PMC6770227

[B24] Zhang Y. Naderi Yeganeh P. Zhang H. Wang S. Y. Li Z. Gu B. (2024). Tumor editing suppresses innate and adaptive antitumor immunity and is reversed by inhibiting DNA methylation. Nat. Immunol. 25 (10), 1858–1870. 10.1038/s41590-024-01932-8 39169233

